# Suicide-related internet use among mental health patients who died by suicide in the UK: a national clinical survey with case–control analysis

**DOI:** 10.1016/j.lanepe.2024.100991

**Published:** 2024-06-28

**Authors:** Lana Bojanić, Pauline Turnbull, Saied Ibrahim, Sandra Flynn, Navneet Kapur, Louis Appleby, Isabelle M. Hunt

**Affiliations:** a National Confidential Inquiry Into Suicide and Safety in Mental Health (NCISH), Centre for Mental Health and Safety, School of Health Sciences, University of Manchester, Manchester, UK; b NIHR Greater Manchester Patient Safety Research Collaboration, Manchester, UK; c Mersey Care NHS Foundation Trust, Liverpool, UK

**Keywords:** Suicide, Internet, Researching suicide methods, Rare suicide methods, Lifespan

## Abstract

**Background:**

Suicide-related internet use (SRIU) has been shown to be linked to suicide. However, there is limited research on SRIU among mental health patients, who are at 4 to 7 times increased risk of suicide compared to the general population. This study aims to address this gap by exploring the prevalence of SRIU among mental health patients who died by suicide in the UK and describing their characteristics.

**Methods:**

The study was carried out as part of the National Confidential Inquiry into Suicide and Safety in Mental Health (NCISH). Data were collected on sociodemographic, clinical, suicide characteristics and engagement in SRIU of patients who died by suicide between 2011 and 2021. The study utilised a case–control design to compare patients who engaged in suicide-related internet use with those who did not.

**Findings:**

The presence or absence of SRIU was known for 9875/17,347 (57%) patients; SRIU was known to be present in 759/9875 (8%) patients. The internet was most often used to obtain information on suicide methods (n = 523/759, 69%) and to visit pro-suicide websites (n = 250/759, 33%) with a significant overlap between the two (n = 152/759, 20%). Engaging in SRIU was present across all age groups. The case–control element of the study showed patients who were known to have engaged in SRIU were more likely to have been diagnosed with autism spectrum disorder (OR = 2.13, 95% CI: 1.43–3.18), have a history of childhood abuse (OR = 1.70, 95% CI: 1.36–2.13) and to have received psychological treatment (OR = 1.43, 95% CI: 1.18–1.74) than controls. Additionally, these patients were more likely to have died on or near a salient date (OR = 2.11, 95% CI: 1.61–2.76), such as a birthday or anniversary.

**Interpretation:**

The findings affirm SRIU as a feature of suicide among patients of all ages and highlight that clinicians should inquire about SRIU during assessments. Importantly, as the most common type of SRIU can expand knowledge on suicide means, clinicians need to be aware of the association between SRIU and choice of methods. This may be particularly relevant for patients approaching a significant calendar event.

**Funding:**

The 10.13039/501100024396Healthcare Quality Improvement Partnership.

**Research in context**

**Evidence before this study**

Research into suicide-related internet use (SRIU) has become a growing focus in suicide prevention. We searched PubMed and PsycINFO databases for research articles in English, published before 1st December 2023, using the search term suicid∗ AND [(internet) OR (online) OR (website)] AND [(psychiatr∗) OR (patient)] as well as grey literature using the same search term in Google Scholar. Finally, we have checked the reference lists of selected articles for further relevant work. The majority of research concerned the general population and specifically young people (i.e., aged under 25 years). The peer-reviewed research concerning suicide-related internet use in mental health patients was cross-sectional and qualitative, weakening the strength of the conclusions and concerned suicidal ideation and self-harm, respectively, rather than death by suicide.

**Added value of this study**

This study used a national consecutive case series of suicides and deaths with undetermined conclusions of people who have been in contact with secondary mental health services within a year before death in the UK, spanning 10 years and including 15,378 patients. Therefore, to the best of our knowledge, this paper gives the first estimate of the prevalence of suicide-related internet use and its various types in a mental health patient population in the UK. A particular strength of this paper in the sub-field of suicide-related internet use research is investigating this behaviour on patients of all ages, rather than focusing on young people as is common in previous work. Additionally, using a case–control analytic approach allowed us to investigate characteristics associated with SRIU in mental health patients more directly. Results show a diagnosis of autism spectrum disorder, recent self-harm, less common methods of suicide and dying on a salient date were more prevalent in those who used the internet for suicide-related purposes compared to patients who died by suicide but did not engage in SRIU.

**Implications of all the available evidence**

SRIU is an important feature of suicide and necessitates more awareness in mental health services. As a behaviour, SRIU can be present in patients of all ages and diagnoses and appears to be associated with the choice of suicide method. Our findings provide evidence in recommending specific inquiry is made by clinical teams into SRIU among patients with mental illness, especially those with autism and recent self-harm.

## Introduction

With over 6000 lives lost to suicide annually^1^ and 98% of the population using the internet,^2^ suicide-related internet use (SRIU) has recently become a key focus in suicide prevention in the UK. SRIU is most commonly defined as ‘use of the internet for reasons relating to an individual's own feelings of suicide’ and can range from being beneficial, such as searching for help and support when feeling suicidal, to harmful use, such as researching suicide methods online.^3^ Recent developments have affirmed SRIU as a recognised factor impacting suicidality, not only by the research and clinical community, but also by legislators and general public stakeholders. The Suicide Prevention Strategy for England^4^ calls for promoting online safety and improved online signposting in order to reduce suicide rates. Additionally, the recent UK Online Safety Act aims to tackle harmful suicide-related online content.^5^

Current estimates of the prevalence of SRIU in the UK general population are mostly based on SRIU in young people. The prevalence of SRIU in a population-based cohort of 21-year-olds was 22.5% and increased three-fold in those who attempted suicide.^6^ In suicide deaths of children and young people aged 10–19 in the UK, evidence of suicide-related online experience (SRIU and online bullying) was present in 24% of deaths, though this high prevalence included exposure to online bullying.^7^ Furthermore, evidence of SRIU was found in 2% of coroners’ reports on possible suicides of adults aged over 25 years in a cross-sectional study in England.^8^ Most SRIU research has focused on this behaviour in the general population,^3^^,^^9^ with current research providing some insight into the prevalence of SRIU in mental health patients as well as their characteristics.

SRIU was associated with 8% of self-harm episodes in hospitalised adults in a UK sample, though this increased over three-fold (26%) in those aged under 18.^10^ In a sample of hospitalised patients with depression, 27% of them reported searching for suicide-related content.^11^ This study also found those who searched for suicide-related content had a more pronounced wish to die, more frequent suicidal ideation, and more previous suicide attempts compared to those who did not engage in SRIU. Finally, a study examining internet searches of hospitalised patients with affective disorders found suicide-related search queries in 63% of patients’ search history.^12^ Searching for methods of suicide seems to feature prominently within SRIU of mental health patients, a potentially risk enhancing behaviour, since reports suggest that up to 54% of webpages with suicide content contain information on highly lethal and less common suicide methods.^13^^,^^14^ Of those who engaged in SRIU and were hospitalised for self-harm, 74% reported searching for suicide methods online.^10^ Additionally, 21% of mental health patients searched for the suicide method that matched the method they had used or planned to use in a suicide attempt.^12^

Increased research focusing on SRIU in mental health patients is important since mental illness is an established risk factor for suicide.^15^ Additionally, current studies suggest that mental health patients use the internet in the same proportion and frequency to the general population.^16^ Finally, their close contact with services offers opportunities for suicide prevention interventions. Therefore, it is important to identify characteristics of patients with SRIU as this could help inform clinical practice and potentially modify clinical care to account for the presence of SRIU. In addition, as the majority of current SRIU research focuses on young people aged under 25, it is important to examine patients’ characteristics and differences across all age groups. The current study aims to address these gaps by exploring SRIU among mental health patients of all ages who died by suicide. The aims were firstly, to describe types of SRIU present in patients who engaged in SRIU and secondly, to describe their socio-demographic, clinical and suicide characteristics and compare these to patients who died by suicide but did not engage in SRIU.

## Methods

### Study design and data source

The study was carried out as part of the National Confidential Inquiry into Suicide and Safety in Mental Health (NCISH). The NCISH methodology has been described in detail elsewhere.^17^ In brief, data collection comprises three stages: (i) collection of all deaths receiving a coroner's conclusion of suicide or undetermined intent from national statistics bodies; (ii) identifying whether the deceased had been in contact with mental health services within 12 months of suicide via administrative contacts in each mental health service provider; and (iii) collection of detailed socio-demographic and clinical information via a questionnaire sent to the clinician responsible for the patient's care. As is conventional in suicide research, deaths with undetermined conclusions are included in the suicide sample.^18^

Since 2011, the NCISH questionnaire included a question on SRIU where it was defined as^1^: obtaining information (e.g. method details) on how to die by suicide^2^; visiting websites that may have discussed/encouraged suicide, including chat rooms (referred to as ‘pro-suicide’ sites)^3^; communicating suicidal ideas online; and^4^ other specified SRIU. Some examples of ‘other’ SRIU included buying means online and researching assisted suicide facilities. Clinicians were able to select one or more types of SRIU that they were aware the patient had engaged in. Additionnaly, clinicians were able to select a ‘not known’ option; this was the case in 43% of patients. For those who died by self-poisoning using opioids, NCISH also collects data on the source of these substances, i.e., prescribed or obtained online without prescription. Patients who had obtained opioids for self-poisoning online without a prescription were included in the final sample of patients with known SRIU. All patients who died by suicide between 1st January 2011 and 31st December 2021 inclusive were included.

NCISH received research ethics approval from North West—GM South REC (reference: ERP/96/136) and Section 251 Approval under the NHS Act 2006 (reference: PIAG 4–08(d)/2003), allowing collection of patient identifiable data for medical research.

### Statistical analysis

Firstly, age comparison between those with recorded presence/absence of SRIU and those without was performed. In subsequent analyses, only patients with recorded presence or absence of SRIU were included, i.e., those with missing values in the SRIU questions were excluded. We examined types of SRIU using frequencies and percentages. Due to the considerable overlap between types of SRIU and small frequencies of some types, SRIU was used as a binary variable in further analyses. To examine trends in suicides of patients with known SRIU, we performed Poisson regression with year of death as a predictor, reporting results as incidence rate ratios (IRR). Due to the delay caused by the time taken to register suicide deaths and the multiple stages of the NCISH methodology, data for trends in the most recent year 2021 were projected based on the number of unreturned questionnaires and the accuracy of the previous year's estimates.^1^ The data used for the rest of the analyses was the actual data collected rather than projected figures. To verify the results, we performed a sensitivity analysis, running Poisson regression without the last year of data (2021) (see Appendix 1).

Descriptive findings of all patient characteristics were presented as proportions and frequencies. The denominators in all estimates were the number of valid cases for the item, i.e., if an item of information was not known for a case, that case was removed from the analysis of that variable. We acknowledge that this could have led to reduced statistical power and underestimation of variability; however, multiple imputation has been deemed unsuitable due to the high percentage of missingness in certain variables. We performed multiple imputation for all variables using chained equations; the direction and significance of the results, albeit with narrower confidence intervals, were confirmed using this approach (see Appendix 2). Subgroup analysis of less common diagnoses and suicide methods (i.e., ‘other’ categories) were not included due to small numbers. Controls (patients who died by suicide and were not known to have engaged in SRIU) were matched by sex and 5-year age bands to cases (patients who engaged in SRIU). All cases were matched with five controls. Conditional logistic regression analysis was used to explore differences between cases and controls, with unadjusted differences presented using conditional odds ratios (ORs) and 95% confidence intervals (CIs). To compensate for multiple comparisons, Bonferroni correction was applied to all p-values; therefore, p-values less than 0.0001 (0.01/number of comparisons) were considered statistically significant. All analyses were performed using Stata version 16.^19^

### Role of the funding source

The study was supported by the Healthcare Quality Improvement Partnership (HQIP) and was carried out as part of the NCISH. All researchers are independent from the funders. The funders of the study had no role in study design, data collection and analysis, interpretation of data, preparation of the manuscript, or decision to publish.

## Results

In the UK between 2011 and 2021, 15,378 patients died by suicide and had been in contact with mental health services in the year before death. The presence or absence of SRIU was known for 9875 (57%) patients, of whom 759 (7.7%) had engaged in SRIU before death. Patients with missing data regarding the presence/absence of SRIU were younger (median age = 44, IQR: 13–96) than those with recorded information on SRIU (median age = 47, IQR: 13–97, p < 0.0001). Suicide trends of the number of patients with known SRIU showed an increase (IRR = 1.07, 95% CI: 1.04–1.09, p < 0.0001) with a peak in 2018, followed by a fall in 2019.

### Types of SRIU

**Fig. 1:**
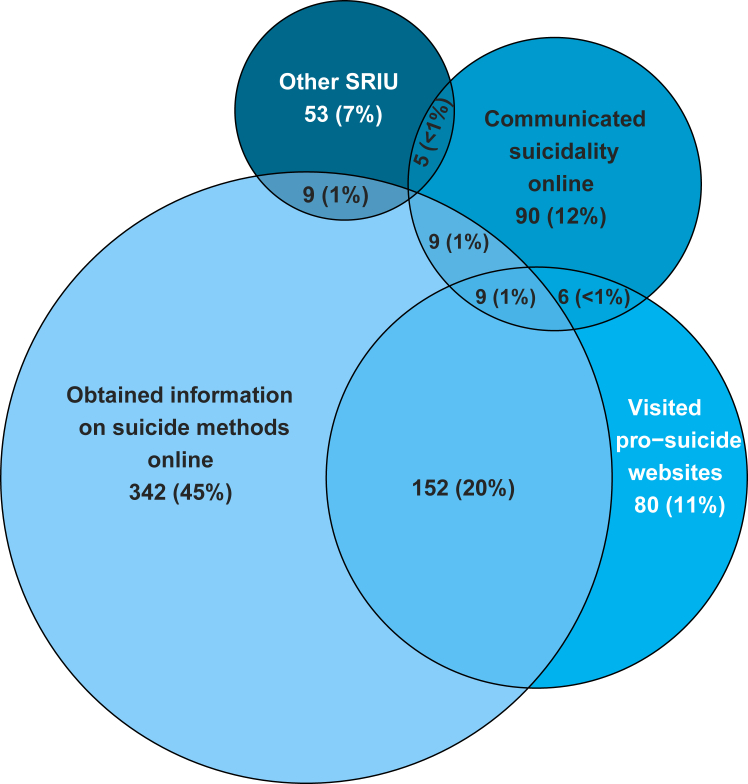
Venn diagram representing frequency and overlap between different types of suicide-related internet use of mental health patients who died by suicide. Note that the information on presence/absence of SRIU was known in 57% of all patients who died by suicide over the study period.

The most common type of SRIU patients engaged in was obtaining information on how to die (n = 523, 68.9%). A third (n = 250, 32.9%) had visited pro-suicide websites and 15.9% (n = 121) had communicated suicidal ideas online. Seventy patients (9.2%) had other types of SRIU, such as obtaining methods online, including four patients who had obtained opioids used in self-poisoning. A quarter of patients had engaged in more than one type of SRIU (25.5%, n = 194). Frequencies and overlaps between different types of SRIU are shown in Fig. 1 (with counts in overlaps of less than 3 not shown).

### Complete sample

In the whole study sample, two-thirds of the patients who engaged in SRIU were male, comparable to other patients (469, 62% v. 5,920, 65%, p = 0.09). Proportions and odds ratios per age group for those who did and did not engage in SRIU are presented in Table 1. Patients who engaged in SRIU were more likely to be younger than 25 and less likely to be aged 45 and above compared to those who did not engage in SRIU. Even though proportionally more patients under 25 engaged in SRIU than in the group with no known SRIU, the majority of the group with any known SRIU was aged between 25 and 64 (73.3%).

**Table 1:** Age groups of mental health patients who died by suicide and who engaged/did not engage in suicide-related internet use (complete sample).

	Any known SRIU N = 759 n (%)	No known SRIU N = 9116 n (%)	Unadjusted OR (95% CI)
**Age group**			
<25	141 (18.6)	701 (7.7)	2.74 (2.25–3.34)^a^
25–44	310 (40.8)	3158 (34.6)	1.30 (1.12–1.51)
45–64	247 (32.5)	3706 (40.7)	0.70 (0.60–0.82)^a^
≥65	61 (8.0)	1551 (17.0)	0.43 (0.33–0.57)^a^

CI: confidence intervals.

a p < 0.0001; Bonferroni correction applied.

Those who engaged in SRIU were more likely to be full-time students (6.4% v. 1.91%), have a primary diagnosis of personality disorder (14.9% v. 10.7%), have any diagnosis of eating disorder (3.1% v. 1.3%), and have had an adverse life event in 3 months before death (51.0% v. 41.1%). They were also more likely to be non-adherent with medication (17.5% v. 12.1%) and die as part of a suicide pact (1.5% v. 0.5%). However, after matching cases with controls, these differences were no longer significant.

### Case-control analysis

Tables 2 and 3 show comparisons between patients who did (cases) and did not (controls) engage in SRIU matched by age and sex. Those who engaged in SRIU were similar in socio-demographics with the exception of being less likely to be unemployed (38.6% v. 51.3%) (Table 2). Compared to controls, those who engaged in SRIU were more likely to have self-harmed in the 3 months before death (42.7% v. 34.5%), and less likely to have both a past and recent history of drug and/or alcohol misuse (46.6% v. 57.8% and 34.3% v. 44.0%, respectively). Patients who engaged in SRIU were more than twice as likely to have been given a diagnosis of autism spectrum disorder (5.0% v. 2.5%), have a history of childhood abuse (27.1% v. 18.5%), and to have received psychological treatment (24.8% v. 18.5%), and were less likely to be diagnosed with schizophrenia (11.0% v. 19.0%) compared to controls (Table 3). Cases and controls differed in methods of suicide: patients who engaged in SRIU were more likely to die by gas inhalation (10.3% v. 1.3%) or self-poisoning (26.3% v. 20.5%), and less likely to die by jumping from a height or in front of a moving vehicle (9.1% v. 16.2%). Finally, patients who engaged in SRIU were more likely to have died on or near a salient date, such as a birthday or an anniversary, compared to controls (14.9% v. 7.8%).

**Table 2:** Socio-demographic, behavioural, and suicide characteristics of patients with (cases) and without (controls) suicide-related internet use who died by suicide.

	Any known SRIU N = 759 n (%)	No known SRIU N = 3,795^d^ n (%)	Unadjusted OR (95% CI)
**Socio-demographic characteristics**			
Ethnic minority group	60 (8.1)	309 (8.3)	0.98 (0.73–1.31)
Unemployed	283 (38.6)	1884 (51.3)	0.58 (0.49–0.69)^e^
Long-term sick	88 (12.0)	413 (11.2)	1.07 (0.83–1.38)
Unmarried	569 (77.3)	2794 (75.5)	1.12 (0.92–1.36)
Living alone	314 (42.5)	1646 (44.1)	0.94 (0.80–1.11)
Full-time student	47 (6.4)	207 (5.6)	1.22 (0.82–1.81)
LGBT+	23 (6.6)	91 (6.2)	1.01 (0.57–1.78)
**Behavioural characteristics**			
History of alcohol and/or drug misuse	341 (46.6)	2149 (57.8)	0.60 (0.50–0.70)^e^
Recent^a^ alcohol and/or drug misuse	247 (34.3)	1620 (44.0)	0.66 (0.55–0.78)^e^
History of self-harm	502 (69.2)	2461 (66.5)	1.13 (0.94–1.35)
Recent^a^ self-harm	306 (42.7)	1271 (34.5)	1.46 (1.23–1.72)^e^
Recently^a^ seen in emergency department for self-harm	184 (27.2)	843 (25.3)	1.09 (0.90–1.32)
History of childhood abuse^b^	150 (27.1)	506 (18.5)	1.70 (1.36–2.13)
Recent^a^ adverse life events^c^	356 (51.0)	1537 (43.9)	1.32 (1.12–1.55)
**Characteristics of suicide**			
Hanging/strangulation	346 (45.7)	1892 (50.1)	0.84 (0.71–0.98)
Self-poisoning	199 (26.3)	776 (20.5)	1.40 (1.17–1.68)^e^
Gas inhalation	78 (10.3)	50 (1.3)	9.27 (6.30–13.64)^e^
Jumping from height/in front of vehicle	69 (9.1)	613 (16.2)	0.51 (0.40–0.67)^e^
Drowning	14 (1.9)	157 (4.2)	0.43 (0.25–0.75)
Suffocation/asphyxiation	34 (4.5)	83 (2.2)	2.08 (1.38–3.12)
Suicide pact	11 (1.5)	14 (0.4)	4.19 (1.88–9.36)
Died on or near a date significant to patient	91 (14.9)	265 (7.8)	2.11 (1.61–2.76)^e^

CI: confidence intervals.

Outcome reference categories: 1- any known SRIU, 0- no known SRIU.

a ’Recent’ refers to 3 months before suicide.

b Includes emotional, physical, and/or sexual abuse.

c Includes divorce, family problems, serious financial difficulties, criminality and violence.

d Matched 5 controls per case.

e p < 0.0001; Bonferroni correction applied.

**Table 3:** Diagnosis and clinical characteristics of patients with (cases) and without (controls) suicide-related internet use who died by suicide.

	Any known SRIU N = 759 n (%)	No known SRIU N = 3,795^c^ n (%)	Unadjusted OR (95% CI)
**Diagnosis**			
Schizophrenia and other delusional disorders	83 (11.0)	711 (19.0)	0.51 (0.40–0.65)^b^
Affective disorder	324 (43.0)	1412 (37.7)	1.27 (1.07–1.50)
Alcohol dependence/misuse	20 (2.7)	224 (6.0)	0.43 (0.27–0.68)
Drug dependence/misuse	15 (2.0)	165 (4.4)	0.44 (0.26–0.76)
Personality disorder	112 (14.9)	517 (13.8)	1.10 (0.87–1.39)
Anxiety disorder	54 (7.2)	160 (4.3)	1.76 (1.28–2.43)
Any^a^ diagnosis of autism spectrum disorder	38 (5.0)	95 (2.5)	2.13 (1.43–3.18)^b^
Any^a^ diagnosis of eating disorder	23 (3.1)	82 (2.2)	1.41 (0.87–2.29)
Any comorbid psychiatric diagnosis	426 (57.0)	2114 (56.6)	1.01 (0.86–1.19)
**Clinical characteristics**			
In-patient	46 (6.1)	342 (9.0)	0.65 (0.47–0.90)
Suicide within 3 months of discharge	112 (15.7)	557 (16.2)	0.97 (0.77–1.21)
Under crisis resolution home treatment services	154 (21.2)	545 (15.3)	1.48 (1.21–1.81)
Missed last appointment	166 (23.7)	752 (22.0)	1.12 (0.92–1.36)
Non-adherent with medication	127 (17.5)	492 (13.4)	1.41 (1.14–1.75)
Receiving any psychological treatment	181 (24.8)	677 (18.5)	1.43 (1.18–1.74)^b^
Receiving any pharmacological medication	639 (85.7)	3249 (86.9)	0.91 (0.72–1.14)
Distressing side-effects of medication	64 (9.8)	248 (8.7)	1.12 (0.84–1.50)
Any comorbid physical illness	153 (20.9)	847 (22.8)	0.89 (0.72–1.09)

CI: confidence intervals.

Outcome reference categories: 1- any known SRIU, 0- no known SRIU.

a Present as either primary, secondary, or tertiary diagnosis.

b p < 0.0001; Bonferroni correction applied.

c Matched 5 controls per case.

## Discussion

Our results suggest that SRIU is a relatively rare behaviour among mental health patients who died by suicide, with an overall prevalence of 8%, though this is likely to be an underestimate. The presence or absence of SRIU was not known in 43% of the patients, indicating that clinicians are not routinely enquiring about internet use. The number of patients who engaged in SRIU based on their clinician's knowledge shows a steady increase with a peak in 2018, corresponding to an overall 2018 rise in patient suicide,^1^ followed by a fall in 2019. The most common types of SRIU were obtaining information on suicide methods and visiting pro-suicide websites, with considerable overlap between the two. Whilst those aged under 25 were more likely to engage in SRIU, we found it was prevalent across all age groups, with the majority being aged 25–44 years. Those who engaged in SRIU were more likely to have a diagnosis of autism spectrum disorder, to have a recent history of self-harm, a history of childhood abuse, and to be receiving psychological treatment at the time of their death. We also found more patients who engaged in SRIU died by gas inhalation or self-poisoning and died on or near a salient date compared to those without SRIU.

To our knowledge, this is the first national in-depth study of SRIU in mental health patients who died by suicide, regardless of age. The rich NCISH dataset has allowed investigation of a wide variety of patient characteristics associated with SRIU, while controlling for age and sex. Matching cases and controls by age has allowed us to compare characteristics irrespective of age, an important consideration since younger age is a commonly discussed factor in SRIU research in general. However, a number of limitations need to be considered. Firstly, this is a clinical population of people in recent contact with secondary mental health services, therefore findings may not be representative of others with mental illness or those in the general population. Even though NCISH questionnaire completeness rate is consistently high (93%) we acknowledge that there are some patients for whom data is not available and we were unable to determine in which way they differ from our study population.^17^ Secondly, without an additional control group of patients with SRIU who did not die by suicide, causal inferences cannot be made. Furthermore, clinicians completing NCISH questionnaires do so based on their knowledge and clinical notes, though most of the information recorded is factual rather than subjective. However, data on the presence or absence of SRIU likely reflects an underestimated prevalence since clinicians do not routinely enquire on SRIU.^20^ Furthermore, there was a high percent of missing data in the variable indicating presence or absence of SRIU; therefore, our prevalence estimate of SRIU should be interpreted with caution. Initial analysis showed that patients for whom there was missing data for the SRIU question were younger, as has previously been reported.^10^ It is possible that the clinicians assumed that older patients did not use the internet thus answering ‘no’ to the question about SRIU. SRIU was used as a binary variable in the analyses. We acknowledge the potential heterogeneity between different types of SRIU, but due to the considerable overlap between some and small frequencies of other types, further investigation was not possible. We did not have information on frequency or duration of time spent engaging in SRIU; nevertheless, we recognise those are potentially important factors. Finally, in this study we were not able to delineate the motivation for patients' SRIU, namely if they have engaged in this behaviour in order to seek support or to facilitate taking their own life. While this study focused on the association between SRIU and suicide thus treating SRIU as a risk factor, the authors acknowledge that SRIU also has a potential in suicide prevention, for example, when searching for help and finding a community to discuss suicide-related problems,^3^ which is currently being explored further by the research team.

We found patients who engaged in SRIU to be considerably older than commonly discussed in SRIU research, which typically focuses on people aged under 25.^6^^,^^7^^,^^9^ This may reflect the fact that more older people have started to use the internet in the UK and therefore more patients have the potential to engage in SRIU.^10^^,^^21^ In addition, the oldest of digital natives, people born in the 1980s and 1990s who grew up with the ubiquitous presence of the internet, are now entering their 30s and 40s. Even though not routinely inquired about in general, qualitative research highlights a tendency for clinicians to inquire about SRIU in young patents only,^20^ potentially missing this risk enhancing behaviour in a significant portion of the patient population.

In line with previous research, we found SRIU was associated with patients dying by gas inhalation and self-poisoning.^22^^,^^23^ Whilst deaths by carbon monoxide poisoning have been steadily declining in the last few decades, the incidence of poisoning with other gasses is on the rise.^22^ SRIU may potentially influence this increase by facilitating both the purchase of these means and instructions for their use. This has also been reported for suicide by self-poisoning, especially with less commonly used substances.^24^ Finally, a higher prevalence of patients with recent self-harm in our sample is in accordance with earlier work.^10^^,^^25^ Biddle and colleagues reported a history of self-harm was more common in young people who searched online for suicide methods and that this type of SRIU was strategic, informing a subsequent suicide attempt.^25^

While numbers were small, we found a diagnosis of autism spectrum disorder (ASD) was more common in those who engaged SRIU. Evidence suggests that autistic people find it easier to communicate and meet people online rather than face-to-face.^26^ In addition, autistic patients are often misdiagnosed and treated by clinicians who do not have experience with childhood onset disorders,^27^ potentially motivating them to turn to the internet for help.^28^ Autistic people are also at a heightened risk of suicide, with population studies indicating suicide risk to be increased between 2.5 and 12 times.^29^ Currently, as the number of mental health patients diagnosed with ASD is rising due to increased clinical recognition of the disorder,^30^ more evidence is needed to discern particular risks of SRIU in these patients.

We found that more patients with SRIU had died around a salient date. While there are numerous reasons why patients would take their lives on or near key dates, it is possible that seeing a social media ‘memory’, a feature that shows content that users had generated or had been tagged in on a certain date, can trigger overwhelming or negative feelings.^31^ However, this warrants further study.

Our results imply that engaging in SRIU is uncommon among mental health patients. However, it is important to note this prevalence estimate stems from clinicians' knowledge on whether the patient engaged in SRIU or not. There is the potential for clinicians overreporting the absence of SRIU when it was not enquired about. Qualitative research conducted by this research team have indicated that currently clinicians do not routinely ask about SRIU.^20^ Therefore, in order to obtain a more accurate picture of patients’ engagement in SRIU before death by suicide, clinicians should inquire about SRIU in all patients, regardless of age. We acknowledge that there may be a need for a different approach to SRIU in a clinical setting depending on other patient characteristics such as having a diagnosis of ASD or engaging in self-harm; however, more research is needed to inform these efforts.

There was a considerable overlap between visiting pro-suicide websites and obtaining suicide methods information online. This potentially points towards a common motivation behind these types of SRIU (i.e., researching suicide methods) or the possibility of one type of SRIU leading to another (i.e., finding pro-suicide websites while obtaining methods information online). Furthermore, there was comparably less overlap between communicating suicidality online and other types of SRIU, suggesting that communicating suicidality online may be a distinct type of SRIU. Delineating between different types of SRIU necessitates further research; however, our results suggest that not all SRIU behaviours are the same and therefore may require a different preventative focus.

The most common type of SRIU in our study concerned searching for suicide methods. This was potentially reflected in differences in suicide methods between patients with and without SRIU. Within the Interpersonal Theory of Suicide, SRIU can potentially increase suicide risk by impacting the capability to engage in suicidal behaviour through exposure to suicide-related content online.^32^ According to the theory, the capability for suicidal behaviour grows through exposure to painful experiences and reduced fear of dying; it is therefore possible that exposure to suicide-related content online can increase one's potential for suicide.^32^ This may be exacerbated by the possible cyclical nature of exposure to suicide-related content in which online algorithms repeatedly recommend to users the content they have searched for.^33^ Searching for methods is an active behaviour, reflecting suicidal planning and could potentially indicate immediate risk and greater availability of means, some with increased lethality.^34^ This again highlights the importance of inquiring about SRIU during assessments. Timely and open invitations to discuss SRIU could interrupt acquiring means and researching suicide by more unusual and less readily available methods. As the internet seems to be a primary source of ‘social contagion’ of emerging methods,^22^^,^^23^ it is important to monitor any mention of novel means of suicide. This could be achieved both at the population level, by regulating the existence and indexing of webpages carrying this information, and at the individual level, steering patients from harmful SRIU and promoting the beneficial components of such internet use.

The implications of this study are timely and relevant, highlighting the need for mental health services to address SRIU among their patients as well as for wider societal responsibility regarding online safety. In clinical settings, the findings underscore the importance of educating clinicians on SRIU and facilitating comprehensive training on SRIU disclosure during patient assessments. Furthermore, the study emphasises the necessity of integrating internet use in patients’ safety planning as well as, more broadly, implementing online safety measures to reduce suicide rates. The study calls for continued research to investigate the potential of SRIU in suicide prevention, and to understand the complex interplay between mental health, the internet, and suicide.

## Contributors

Lana Bojanić conceptualised the research question, curated the data, conducted formal analysis, designed the figure, interpreted the data and wrote the original draft. Dr Saied Ibrahim verified the formal analysis and Dr Isabelle M Hunt interpreted the data alongside the first author. All authors contributed equally to the review and editing and were jointly responsible for the decision to submit the manuscript.

## Data sharing statement

Electronic health records are, by definition, considered sensitive data in the UK by the Data Protection Act and cannot be shared via public deposition because of information governance restrictions in place to protect patient confidentiality.

## Declaration of interests

N.K. is a member of the Department of Health and Social Care (England) National Suicide Prevention Advisory Group. He chaired the NICE Guideline Development Group for the Longer-Term Management of Self-Harm and the NICE Topic Expert Group (which developed the quality standards for self-harm services). N.K. is currently chair of the updated NICE Guideline for Depression and topic advisor for the current NICE Guideline Development Group for the Longer-term Management of Self-harm and is also supported by Mersey Care NHS Foundation Trust. L.A. is Chair of the National Suicide Prevention Strategy Advisory Group, DHSC. N.K., L.A. and P.T. report grants from the Healthcare Quality Improvement Partnership and NHS England. N.K., L.A. and P.T. report grants from the Department for Education and the Medical Research Council. P.T. reports a grant from the Gambling Research Exchange Ontario. I.H. reports grants from HQIP and from the Medical Protection Society Limited (MPS). All other authors declare no competing interests.
