# Supplementary material for: Suicide-related internet use among mental health patients who died by suicide in the UK: a national clinical survey with case–control analysis
Source: Lancet Reg Health Eur. 2024 Jun 28;44:100991. doi: 10.1016/j.lanepe.2024.100991 (PMC11262162; doi:10.1016/j.lanepe.2024.100991)
Supplement: Appendix 1 [file mmc1.docx]

Appendix 1: Poisson regression

Poisson regression was carried out with the dependent variable being the count of patients who had engaged in SRIU and the independent variable being the year of death. The regression equation is as follows:

log(λ_i_​)=β_0_​+β_1_×_​_Year_i_

Here, λ_i_ is the expected number of patients who died by suicide and engaged in SRIU for every year (i), β_0_​ is the intercept, and β_1​_ is the coefficient for the predictor variable (Year_i_). To verify the results, we performed a sensitivity analysis, running Poisson regression without the last year of data (2021). Results of both Poisson regressions are presented in Table 1.

Table 1. Poisson regressions results for the number of patients with suicide-related internet use by year of death (Model 1: years 2011-2021, Model 2: years 2011-2020).

|  | **Model 1 (2011-2021)**  **IRR (95% CI)** | **Model 2 (2011-2020)**  **IRR (95% CI)** |
| --- | --- | --- |
| Intercept | 7.4 ×10^−59^ (3.7 ×10^−79­^—1.4 ×10^−38^) | 2.2 ×10^−71^ (2.7 ×10^−94­^—1.8 ×10^−48^) |
| Year | 1.07 (1.04-1.09)* | 1.08 (1.05-1.11)* |
| *IRR: incident rate ratio*  *CI: confidence intervals*  **p<0.0001: Bonferroni correction applied* | | |

The trend in SRIU over time is shown in Figure 1. Due to the delay caused by the time taken to register suicide deaths and the multiple stages of the NCISH methodology, data for trends in the most recent year 2021 were projected based on the number of unreturned questionnaires and the accuracy of the previous year’s estimates.

Figure 1. Frequency of suicide-related internet use in patients who died by suicide between 2011 and 2021.
