# Supplementary material for: Suicide-related internet use among mental health patients who died by suicide in the UK: a national clinical survey with case–control analysis
Source: Lancet Reg Health Eur. 2024 Jun 28;44:100991. doi: 10.1016/j.lanepe.2024.100991 (PMC11262162; doi:10.1016/j.lanepe.2024.100991)
Supplement: Appendix 2 [file mmc2.docx]

Appendix 2. Multiple imputation- sensitivity analysis

We performed multiple imputation using chained equations (MICE); this analysis uses a different conditional distribution for the imputation of each variable. The imputation was carried out prior to case-control matching. Five multiply imputed datasets have been created and the results were pooled using Rubin's rules to produce pooled estimates. The analysis was performed using Stata version 16. Results of the conditional logistic regression are presented in Tables 1 and 2.

Table 1. Socio-demographic, behavioural, and suicide characteristics of patients with (cases) and without (controls) suicide-related internet use who died by suicide: non-imputed and multiply imputed dataset.

|  | **Unadjusted OR**  **(95% CI)** | **Unadjusted OR**  **(95% CI)**  **Multiple imputation** |
| --- | --- | --- |
| **Socio-demographic characteristics** |  |  |
| Ethnic minority group | 0.98 (0.73-1.31) | 0.98 (0.74-1.31) |
| Unemployed | 0.58 (0.49-0.69)* | 0.59 (0.50-0.70)* |
| Long-term sick | 1.07 (0.83-1.38) | 1.07 (0.83-1.37) |
| Unmarried | 1.12 (0.92-1.36) | 1.12 (0.92-1.37) |
| Living alone | 0.94 (0.80-1.11) | 0.95 (0.81-1.12) |
| Full-time student | 1.22 (0.82-1.81) | 1.21 (0.81-1.81) |
| LGBT+ | 1.01 (0.57-1.78) | 1.27 (0.80-2.04) |
| **Behavioural characteristics** |  |  |
| History of alcohol and/or drug misuse | 0.60 (0.50-0.70)* | 0.60 (0.51-0.71)* |
| Recent^1^ alcohol and/or drug misuse | 0.66 (0.55-0.78)* | 0.65 (0.55-0.78)* |
| History of self-harm | 1.13 (0.94-1.35) | 1.14 (0.95-1.37) |
| Recent^1^ self-harm | 1.46 (1.23-1.72)* | 1.45 (1.22-1.73)* |
| Recently^1^ seen in emergency department for self-harm | 1.09 (0.90-1.32) | 1.03 (0.86-1.24) |
| History of childhood abuse^2^ | 1.70 (1.36-2.13)* | 1.49 (1.17-1.88) |
| Recent^1^ adverse life events^3^ | 1.32 (1.12-1.55) | 1.32 (1.13-1.56) |
| **Characteristics of suicide** |  |  |
| Hanging/strangulation | 0.84 (0.71-0.98) | 0.83 (0.71-0.98) |
| Self-poisoning | 1.40 (1.17-1.68)* | 1.40 (1.16-1.68)* |
| Gas inhalation | 9.27 (6.30-13.64)* | 9.21 (6.26-13.56)* |
| Jumping from height/in front of vehicle | 0.51 (0.40-0.67)* | 0.52 (0.40-0.67)* |
| Drowning | 0.43 (0.25-0.75) | 0.43 (0.25-0.76) |
| Suffocation/asphyxiation | 2.08 (1.38-3.12) | 2.09 (1.39-3.14) |
| Suicide pact | 4.19 (1.88-9.36) | 4.53 (1.96-10.48) |
| Died on or near a date significant to patient | 2.11 (1.61-2.76)* | 2.15 (1.69-2.74)* |

Table 2. Diagnosis and clinical characteristics of patients with (cases) and without (controls) suicide-related internet use who died by suicide: non-imputed and multiply imputed dataset.

|  | **Unadjusted OR**  **(95% CI)** | **Unadjusted OR**  **(95% CI)**  **Multiple imputation** |
| --- | --- | --- |
| **Diagnosis** |  |  |
| Schizophrenia and other delusional disorders | 0.51 (0.40-0.65)* | 0.51 (0.39-0.65)* |
| Affective disorder | 1.27 (1.07-1.50) | 1.27 (1.08-1.50) |
| Alcohol dependence/misuse | 0.43 (0.27-0.68) | 0.43 (0.27-0.69) |
| Drug dependence/misuse | 0.44 (0.26-0.76) | 0.44 (0.26-0.74) |
| Personality disorder | 1.10 (0.87-1.39) | 1.11 (0.88-1.40) |
| Anxiety disorder | 1.76 (1.28-2.43) | 1.72 (1.25-2.37) |
| Any^1^ diagnosis of autism spectrum disorder | 2.13 (1.43-3.18)* | 2.12 (1.42-3.17)* |
| Any^1^ diagnosis of eating disorder | 1.41 (0.87-2.29) | 1.41 (0.87-2.28) |
| Any comorbid psychiatric diagnosis | 1.01 (0.86-1.19) | 1.02 (0.87-1.19) |
| **Clinical characteristics** |  |  |
| In-patient | 0.65 (0.47-0.90) | 0.65 (0.47-0.90) |
| Suicide within 3 months of discharge | 0.97 (0.77-1.21) | 0.94 (0.74-1.19) |
| Under crisis resolution home treatment services | 1.48 (1.21-1.81) | 1.53 (1.25-1.86) |
| Missed last appointment | 1.12 (0.92-1.36) | 1.12 (0.92-1.36) |
| Non-adherent with medication | 1.41 (1.14-1.75) | 1.38 (1.11-1.71) |
| Receiving any psychological treatment | 1.43 (1.18-1.74)* | 1.43 (1.19-1.73)* |
| Receiving any pharmacological medication | 0.91 (0.72-1.14) | 0.91 (0.72-1.14) |
| Distressing side-effects of medication | 1.12 (0.84-1.50) | 1.12 (0.84-1.51) |
| Any comorbid physical illness | 0.89 (0.72-1.09) | 0.88 (0.72-1.07) |
